# An updated review of epidemiological characteristics, immune escape, and therapeutic advances of SARS-CoV-2 Omicron XBB.1.5 and other mutants

**DOI:** 10.3389/fcimb.2023.1297078

**Published:** 2023-12-14

**Authors:** Zongming Liu, Jiaxuan Li, Shanshan Pei, Ying Lu, Chaonan Li, Jiajie Zhu, Ruyi Chen, Di Wang, Jingbo Sun, Keda Chen

**Affiliations:** ^1^ Shulan International Medical College, Zhejiang Shuren University, Hangzhou, China; ^2^ Sir Run Shaw Hospital, School of Medicine, Zhejiang University, Hangzhou, China; ^3^ School of Pharmacy, Beihua University, Jilin, China

**Keywords:** Omicron XBB.1.5, epidemiological characteristics, immune escape, treatment strategies, SARS- CoV- 2

## Abstract

The rapid evolution of Severe Acute Respiratory Syndrome Coronavirus 2 (SARS-CoV-2) has led to the emergence of new variants with different genetic profiles, with important implications for public health. The continued emergence of new variants with unique genetic features and potential changes in biological properties poses significant challenges to public health strategies, vaccine development, and therapeutic interventions. Omicron variants have attracted particular attention due to their rapid spread and numerous mutations in key viral proteins. This review aims to provide an updated and comprehensive assessment of the epidemiological characteristics, immune escape potential, and therapeutic advances of the SARS-CoV-2 Omicron XBB.1.5 variant, as well as other variants.

## Introduction

**Figure 1:**
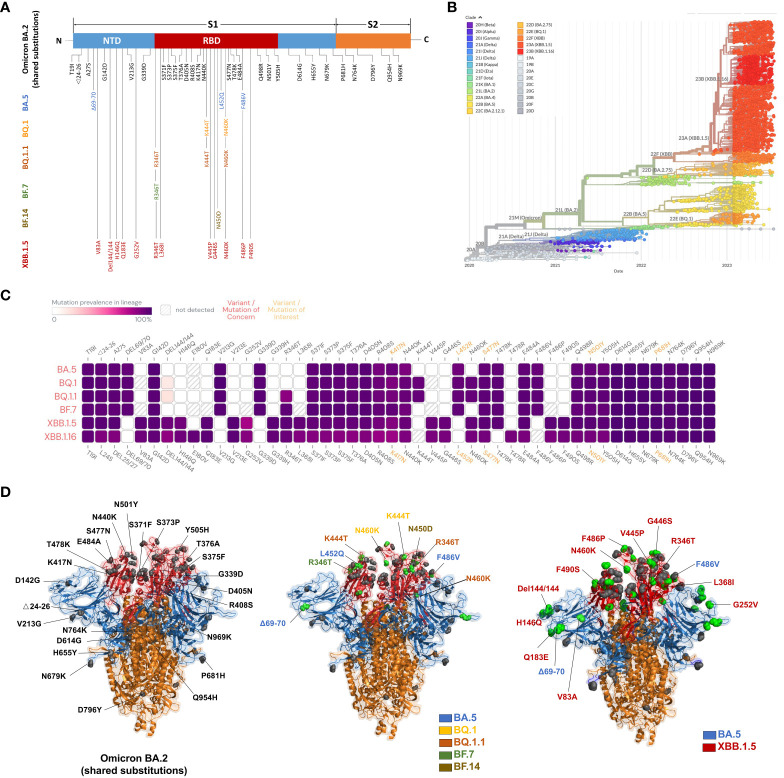
**(A)** Time-scale phylogenetic tree of a representative global subsample of the SARS-CoV-2 genome, colored according to cues from Nextstrain clades that correspond primarily to variants of interest. Figure modified from Nextstrain’s “Omicron-recombinant” construct (2023-8-12). **(B)** Amino acid substitutions in Omicron mutant strains. Black represents the Omicron BA.2 mutation site, which is also a shared mutation. Blue represents Omicron BA.5, yellow represents BQ.1, orange represents BQ.1.1, green represents BF.7, brown represents BF.14, and red represents XBB.1.5. Figure was drawn using Microsoft Office PowerPoint. **(C)** Mutation prevalence across lineages. The Outbreak platform provides S protein mutation prevalence across lineages. The figure describes in detail the mutation prevalence in the lineage of VOCs. Orange indicates a mutation of interest, and red indicates a mutation of concern. Blank indicates that the mutation has not been detected. White to purple indicates the prevalence of the mutation in all sequences. **(D)** Full-length spiking proteins were constructed based on relative mutation sites (3D structure). Black represents the Omicron BA.2 mutation site, which is also a shared mutation site. Blue represents Omicron BA.5, yellow represents BQ.1, orange represents BQ.1.1, green represents BF.7, brown represents BF.14, and red represents XBB.1.5. Figure by Pymol; residues are from PDB (www.rcsb.com).

Continuous mutations of severe acute respiratory syndrome coronavirus 2 (SARS-CoV-2) lead to the continued occurrence of breakthrough infections with coronavirus disease 2019 (COVID-19) (Cao et al., 2022). As of July 2022, countries around the world are experiencing a new wave of SARS-CoV-2 infections, primarily driven by the Omicron BA.5 variant, which was initially detected in nasal swabs from South African patients (Tegally et al., 2022b). Recently, three new subvariants of Omicron, namely BF.7, BQ.1, and XBB, have gradually replaced other Omicron subvariants in many countries, drawing global attention. It’s noteworthy that BF.7, BQ.1, XBB, and their sub-lineages exhibit stronger transmissibility and immune escape potential, posing significant challenges to epidemic prevention and control (**Figure 1A**). Regarding the disease symptoms caused by the Omicron variant, there are some reasons for optimism. Early evidence suggests that a higher proportion of patients infected with Omicron experience mild symptoms, and hospitalization rates may be lower compared to other SARS-CoV-2 variants (Jassat et al., 2022a; Jassat et al., 2022b). Whether current vaccines and serum from people who have recovered from breakthrough infection can provide sufficient protection against Omicron mutants, and whether existing drugs are effective against COVID-19, has become a common concern among the public (Mohapatra et al., 2022).

Given the potential impact of variants like Omicron XBB.1.5 and other mutants on global epidemic prevention and control, it is crucial to gain a deeper understanding of their transmission and pathogenic characteristics, clinical symptoms, immune evasion mechanisms, and treatment strategies. By synthesizing existing evidence, this review seeks to inform public health decision-makers, healthcare professionals, and the public about the current state of knowledge regarding subvariants like Omicron XBB.1.5 and guide the formulation of effective strategies to address ongoing pandemics.

## Evolution of the Omicron variants

Since its initial report after 2021, Omicron variants have emerged in multiple sub-lineages that vary in the number of mutations and levels of infectivity (Alam, 2023). BA.1.1 is the first subline with specific mutations, such as R346K in the S protein that causes immune evasion (Mannar et al., 2022). BA.2 has novel mutations, including T376A, L452, F486, and R408S, which also confer immune evasion (Martin et al., 2022). BA.2.12.1 and BA.2.13 have L452Q and L452 M specific mutations respectively, and both have more significant transmission advantages than BA.2 (Dhawan et al., 2022b). Both BA.4 and BA.5 have the L452R+F486V mutation (Dhawan et al., 2022a). The S protein mutation at L452R is thought to be responsible for increased transmissibility. Prior research has elucidated that both BA.4 and BA.5 share an identical S sequence, showcasing a heightened affinity for binding to the ACE2 receptor. Moreover, these subvariants encompass distinctive mutations, notably encompassing Del69-70, L452R, F486V, and R493Q. It has been observed that Del69-70, L452R, and F486V have the capacity to incite antibody resistance, whereas R493Q confers susceptibility to antibody interactions (**Figures 1B-D**). After this, BA.5 underwent further evolutionary changes, leading to the emergence of BA.5.2.1.7, commonly referred to as BF.7 (Akif et al., 2023). Based on the information accessible through reports, a discernible trend has emerged indicating a direct correlation between the immune evasion capacity of the BF.7 variant and the specific occurrence of the R346T mutation within the spike protein of the SARS-CoV-2 viral strain (Varghese et al., 2023). BQ.1 and BQ.1.1 have arisen as derivatives stemming from the BA.5 lineage. Notably, both BQ.1 and BQ.1.1 exhibit analogous mutations within the spike protein inherent to the BA.5 variant. In conjunction with these shared mutations, BQ.1 carries supplementary genetic alterations, including K444T, N460K, and Q493R, while BQ.1.1 encompasses an additional substitution denoted as R346T. The existence of mutations, exemplified by L452R and F486V, has resulted in a twofold augmentation in the infective potential of the BA.5 variant when juxtaposed with its predecessor, BA.2. As a result, this escalated infectivity has expedited the replacement of the BA.2 variant by BA.5, a transition that was completed by the conclusion of June 2022 (Chen et al., 2022). These mutations have been inherited by the BQ.1 and BQ.1.1 lineages, thereby conferring an additional advantage in terms of transmission. The binding free energy demonstrated by the complex formed between the receptor binding domain (RBD) of BQ.1.1 and the hACE2 exceeds 4.0 kcal/mol, surpassing the values observed in both the BQ.1 and BA.5 variants (Chen et al., 2022). This indicates a high likelihood that the Omicron variant, referred to as XBB.1.5, is poised to replace the previous BA.5 variant, aligning with the observed rapid spread witnessed within the context of BQ.1.1. XBB.1.5 is identified as a hybrid iteration originating from the preexisting variants, BA.2.10.1 and BA.2.75. Notably, most mutations occurring within the spike protein can be attributed to the evolutionary lineage of BA.2. The genetic composition of the Omicron variant is characterized by the emergence of ten novel mutations, designated as V83A, H146Q, Q183E, V213E, G339H, R346T, L368I, F486S, and F490S, in conjunction with the presence of an amino acid deletion at position 144. The variants XBB.1 and XBB.1.5 display an additional substitution, specifically G252V, in comparison to the XBB reference strain. Furthermore, XBB.1.5 is distinguished by the inclusion of an F486P substitution, diverging from the F486S mutation identified in XBB and XBB.1. This comprehensive array of mutational changes serves as a fundamental framework for elucidating the genetic evolution of the Omicron variant. In juxtaposition to the progenitor strains, this infrequent substitution (F486P) demonstrates a discernible correlation with alterations in both the binding affinity and transmissibility of the RBD to the hACE2 (Yue et al., 2023). Experiments using lentivirus-based pseudoviruses also showed approximately 3-fold increased infectivity of XBB.1.5 compared with XBB.1. These results suggest that XBB.1.5 exhibits a remarkably strong affinity to the human ACE2 receptor, which is attributed to the S486P substitution (Uriu et al., 2023).

New research examines the alterations in the electrostatic potential of the receptor-binding domain (RBD) of the SARS-CoV-2 S protein in Omicron variants in comparison to Delta and Delta-plus variants. The researchers suggest that the increased positive electrostatic potential of the Omicron variant could enhance its transmissibility. They conducted an analysis to assess the influence of Omicron variant VOC mutations on the interaction between the S protein and ACE2 receptor (Pascarella et al., 2022). The findings indicate a significant rise in the positive electrostatic potential within the binding domain of the RBD interface with ACE2 in the Omicron variant. This augmentation in positive electric potential may intensify the affinity of the viral S protein for host cells, potentially elucidating the higher transmissibility of Omicron variants (Pawłowski, 2021). However, the researchers acknowledge that the actual *in vivo* effects might be influenced by the presence of other mutations and potential alterations in interaction patterns with ACE2 and other macromolecules. Therefore, further studies are necessary to comprehensively comprehend the impact of these changes on the transmissibility of Omicron variants.

## Epidemiological characteristics of Omicron subtype

In accordance with the guidelines provided by the European Centre for Disease Prevention and Control (ECDC), the probability of hospitalization or fatality subsequent to SARS-CoV-2 infection exhibits a discernible association with progressive age and underlying health morbidities. These health conditions encompass chronic obstructive pulmonary disease (COPD) or other chronic respiratory ailments, cardiovascular afflictions, persistent kidney disorders, states of immunodeficiency, diabetes, obesity, hypertension, solid organ and hematologic malignancies, in addition to neurological dysfunctions (National Center for Immunization and Respiratory Diseases (NCIRD), Division of Viral Diseases (2022); Bellou et al., 2022). The clinical classification of individuals afflicted by the virus primarily encompasses those exhibiting mild and asymptomatic manifestations, and they tend to belong to a relatively higher median age bracket. It is noteworthy to mention that infections attributed to the BA.4/5 variant are characterized by more pronounced and widespread symptomatic presentations, encompassing, but not confined to, symptoms such as fatigue, cough, fever, and headache. The duration of these symptoms persists for a duration of four days, which is in contradistinction to the seven-day duration observed in cases of BA.1 infections.

Bayesian analysis elucidates that the effective reproductive number (Re) pertaining to BA.4/5 exceeds that corresponding to BA.2 (Kimura et al., 2022c). The latent period associated with Omicron variant BA.5 infection is conspicuously brief, spanning a range of 2 to 3 days. Moreover, it exhibits a growth advantage over the BA.2 variant by a magnitude of 0.10, accompanied by a 95% confidence interval ranging from 0.09 to 0.11. The foundational reproduction number (R0), quantified at 18.6, draws a marked parallel to the transmission characteristics of the highly communicable measles virus (R0 values ranging from 12 to 18) (Tegally et al., 2022a). The study outcomes unveil an adjusted hazard ratio [aHR] of 1.12 (with a 95% confidence interval of 0.93 to 1.34) concerning the susceptibility to hospitalization, severe morbidity, and fatality among individuals afflicted by the BA.5 variant in both South Africa and the United States during the temporal span ranging from April to September 2022(Tian et al., 2022). Furthermore, Kimura et al. have unveiled a substantial disparity, noting that the relative replication rate (Re) of BA.5 surpasses that of BA.2 by a factor of 21. Additionally, BA.5 demonstrates enhanced infectivity towards human lung cells and golden hamsters, in comparison with BA.2. Moreover, it is important to highlight that the pseudovirus infectivity of the Omicron variant BA.5 significantly outpaces that of BA.2, exhibiting an 18.3-fold increase (Kimura et al., 2022b).

In contrast to the antecedent Omicron variant, characterized by an average basic reproduction number (R0) of 5.08, the BF.7 variant exhibits a conspicuously heightened infectivity profile, spanning an R0 spectrum ranging from 10 to 18.6. This escalated infectivity profile gives rise to the distinct attributes of rapid transmission kinetics and a truncated incubation period within the BF.7 variant. Furthermore, it demonstrates an augmented predisposition to affect individuals previously identified as having tested positive for COVID-19, as well as those who have undergone vaccination, or even those who have received dual vaccination regimens (Varghese et al., 2023).

Upon adjusting the Reynolds number (Re) to 1 for BA.5, a conspicuous outcome emerges: the relative Reynolds values associated with BQ.1 and BQ.1.1 stand at 1.23 and 1.24, correspondingly. This discernible pattern accentuates the augmented propagation propensity exhibited by BQ.1, distinctly juxtaposed against the characteristics of BA.2 and BA.5, all within the confines of the specified parameters (Tamura et al., 2023). The geographic scope of viral propagation experienced a swift and conspicuous expansion, leading to the displacement of the formerly predominant BA.5 lineage across multiple nations. As of mid-September 2022, the incidence of infections attributed to the BQ.1 and BQ.1.1 variants constituted approximately 2% of the cumulative reported global cases. However, this proportion underwent a substantial escalation, surging to 52.22% by early December 2022. In the mid-December of 2022, the viral strains denoted as BQ.1 and BQ.1.1 were responsible for 60.92% and 60% of the documented cases of infection in the territorial boundaries of the United States and the United Kingdom, correspondingly. Subsequent to this period, by January 2023, these variants had achieved a prevalence surpassing the 50% benchmark on a worldwide scale (Ao et al., 2023).

Following this, the rapid proliferation of XBB.1.5 occurred throughout the United States. Reputable scholarly inquiries assert that when establishing the Reynolds number (Re) of BA.5 as 1, the ensuing relative Reynolds numbers for XBB and XBB.1 are determined to be 1.24 and 1.26, respectively (Tamura et al., 2023), The Reynolds number (Re) of XBB.1.5 surpasses that of XBB.1 by a factor exceeding 1.2 (Uriu et al., 2023), This observation denotes an augmentation in infectivity, an expedited propagation rate, and an elevated proficiency in evading immune responses within XBB.1.5, when juxtaposed with its counterparts existing within the Omicron sublineages. It is noteworthy that this trend is particularly conspicuous within demographic cohorts devoid of prior exposure to Omicron variants (Akif et al., 2023). According to evaluations conducted by the United States Centers for Disease Control and Prevention (CDC), as of April 29, 2023, the XBB.1.5 variant accounts for 73.5% of the recently documented instances of infection within the territorial boundaries of the United States (Prevention Centers for Disease Control and. COVID Data Tracker. Retrieved from https://covid.cdc.gov/covid-data-tracker/#variant-proportions). Based on the report issued by the World Health Organization, as of 8 August 2023, a cumulative count of 272,942 instances of the XBB.1.5 subvariant were formally documented across a spectrum of 120 nations, and the strain has been detected in at least 54 U.S. states. SARS-CoV-2 (hCoV-19) Mutation Reports. Retrieved from https://outbreak.info/situation-reports

## Omicron variants are highly resistant to convalescent serum from previous infection

A comprehensive study examined whether prior infections with different SARS-CoV-2 variants confer protection against subsequent reinfections (Dangi et al., 2021). It was found that prior exposure to variants like Alpha, Beta, or Delta provides a degree of immunity against BA.4 or BA.5 reinfections, with Omicron infections offering even stronger protection (Prillaman, 2022). Serum samples from individuals who had breakthrough infection by Delta, BA.1, BA.2.2, BA.5.12, and BA.2.76 showed varying degrees of immune evasion when exposed to neutralizing antibodies against BQ.1 and BQ.1.1 (Jiang et al., 2023). Breakthrough infections involving BA.1 notably reduced the serum’s ability to neutralize BQ.1.1, compared to higher rates for BA.2 (94.7%) and BA.2.75 (100%) (Jiang et al., 2023). Strong immune evasion by both BQ.1 and BQ.1.1 when tested against serum from convalescent individuals linked to BA.1 and BA.4/5 variants, surpassing neutralization resistance exhibited by other variants (Qu et al., 2023a). In individuals who received three doses of mRNA vaccines and later contracted BA.2 infections, their neutralizing capacities against BQ.1.1 and the XBB subvariants were significantly diminished by 35.2-fold and 61.7-fold, respectively, compared to the ancestral strain (Uraki et al., 2023a). Moreover, XBB.1 and XBB.3 exhibited greater immune escape potential compared to the wild type in patients with varying vaccination histories and doses (Zhang et al., 2023). Serum from convalescent individuals who received the three-dose CoronaVac regimen before encountering D614G, BA.5, or BF.7 variants showed notable protection against XBB.1.5(Liu et al., 2023). However, breakthrough infections consistently showed reduced neutralizing titers against XBB.1 and XBB.1.5. In cases of BA.5 or BF.7 infections following CoronaVac vaccination, neutralizing titers against XBB.1.5 decreased significantly, ranging from 27 to 40 times lower (Qu et al., 2023b; Qu et al., 2023c) (**Table 1**).

**Table 1:** Therapeutic efficacy of convalescent serum, vaccine and existing COVID-19 drugs against SARS-CoV-2 mutant strain (Bruel et al., 2022; Qu et al., 2022; Takashita et al., 2022; Cho et al., 2023; Lasrado et al., 2023; Zhu et al., 2023).

Convalescent Serum\Vaccine\ Drugs		BA.2	BA.5	BF.7	BQ.1	BQ.1.1	XBB.1.5
WH- infection		32	37	31	30	30	30
Delta- infection		41	42	35	31	31	31
BA.1- infection		439	263	227	135	145	/
BA.4/5- infection		341	190	162	68	66	/
BA.5- infection		188	445	121	109	60	31
CoronaVac*3		31	32	30	30	30	30
BNT162b2*3		271	213	83	52	45	35
ChAdOx1*3		130	72	/	27	24	/
CoronaVac*2+ BNT162b2*1		110	133	70	52	43	32
CoronaVac*2+ Ad5-nCoV*1		329	375	174	161	121	43
mRNA-1273*3 or mRNA-1273*2+ BNT162b2*1		759	300	238	140	114	/
BNT162b2*4 or mRNA-1273*4		88	62.2	/	/	16.8	/
Spike‐XBB vaccine		/	1795	969	688	502	3700
Spike‐XBB.1.5 vaccine		/	3148	1405	1151	938	6728
Antiviral Drug (IC50; μM)	Remdesivir	2.77	1.91	2.45	/	2.0	2.19
	Nirmatrelvir	2.07	0.51	0.85	/	1.30	1.20
	Molnupiravir	8.09	1.94	9.4	/	6.62	5.18
Monoclonal Antibody (IC50 (ng/ML))	Sotrovimab	>9,000	1,088	>2000	600-1709	> 10000	> 10000
	Cilgavimab	6.1	11	>2000	>10000	> 10000	> 10000
	Bebtelovimab	4.5	2	/	>10000	> 10000	> 10000
	Casirivimab	>9,000	>9,000	>9,000	> 10000	> 10000	> 10000
Protein Inhibitor	Nirmatrelvir	6.9μmol	4.4μmol	10.8μmol	/	/	/

The results of the mice experiments are shown in blue.

“/” No relevant data found, “*” The number of doses of the vaccine administered.

## Omicron’s capacity for immune evasion from neutralizing antibodies induced by vaccines

The neutralization efficacy of the tripartite CoronaVac vaccine against BQ.1, BQ.1.1, XBB, and XBB.1 demonstrated a prominent reduction (Cao et al., 2023). Research endeavored to conduct conducted a comprehensive assessment of the neutralizing activity present in sera derived from individuals who had received a three-dose regimen of the BNT162b2 mRNA vaccine or had encountered infection with the BA.1 variant. The findings have brought to light that the neutralizing efficacy of sera originating from individuals subjected to the three-dose BNT162b2 mRNA regimen displayed a conspicuous reduction when directed towards the BF.7, BQ.1, BQ.1.1, and XBB.1.5 subvariants, in comparison to their performance against the original strain (Kurhade et al., 2022b). Sera obtained from healthcare workers who have completed a three-dose regimen of mRNA vaccine exhibit robust neutralizing efficacy against the BQ.1 and BQ.1.1 subvariants (Arunachalam et al., 2023; Tauzin et al., 2023). The scrutiny of neutralization titers elucidates that when compared to the D614G variant, the neutralizing potency of serum antibodies against BA.4/5, BF.7, BQ.1, and BQ.1.1 underwent decrements by factors of 8.72, 11, 18.7, and 22.9, respectively (Qu et al., 2023a). A meticulous examination of the protective effectiveness induced by varying dosing regimens of BNT162b2 or mRNA-1273 vaccines against the BQ.1/BQ.1.1 and XBB/XBB.1 variant underscores that the immunogenic response triggered by the application of three or four vaccine doses persists at a negligible level, virtually evading detection (Uraki et al., 2023a). During the Omicron pandemic, Moderna embarked on the formulation of a binary vaccine aimed at the Omicron variant, prompting inquiries into the efficacy of the resultant bivalent BA.5 vaccine about its performance against BQ.1 and XBB (Miller et al., 2023). Studies have shown that vaccination with the BA.5 bivalent booster can induce high neutralizing titers against BA.4/5 14-32 days after boosting, but it does not produce antibodies against BA.2.75.2, BQ.1.1 or XBB.1 high level of neutralizing effect (Kurhade et al., 2022a). The findings unequivocally highlighted that the administration of a four-dose mRNA vaccine regimen failed to elicit efficacious preventive effects against the emerging Omicron subvariants. Notably, the NAb for the XBB.1 sublineage demonstrated a conspicuous nadir, falling below the established detection threshold (<20). The BQ.1.1 subvariant exhibited a similar trend. Nevertheless, the introduction of the bivalent BA.5 booster exhibited a distinct yet statistically non-significant augmentation in neutralization capacity against all subsequent Omicron iterations. NAb titers against BA.4/5, BF.7, BA.4.6, BA.2.75.2, BQ.1.1 and XBB.1 in sera from subjects who received a bivalent BA.5 boost but had no history of infection are 298, 305, 183, 98, 73 and 35 respectively, while the corresponding measurement of WT is 3620 (Kurhade et al., 2022a). Aligned with this concept, in comparison to alternative vaccine formulations, the protocol involving a bivalent booster demonstrates an elevated degree of effectiveness against XBB. It is noteworthy to emphasize, however, that the magnitude of immune augmentation attained remains notably restrained (Davis-Gardner et al., 2022). A comprehensive study was conducted to thoroughly evaluate the immune response among participants who received a bivalent booster vaccine at three distinct time points. The findings revealed that both the XBB and XBB.1.5 variants exhibit the ability to elude neutralizing antibody (NAb) responses, while not displaying an equivalent evasion of T-cell responses. Importantly, the NAb titers observed for XBB.1 and XBB.1.5 are comparable, suggesting that the F486P mutation within XBB.1.5 could potentially heighten its transmissibility without concomitantly augmenting its capacity to evade the immune system(Lasrado et al., 2023) (**Table 1**).

## Omicron demonstrates immune evasion against therapeutic monoclonal antibodies

Amid the evolving landscape of SARS-CoV-2 mutations, a succession of clinically authorized therapeutic monoclonal antibodies (bamlanivimab, etesevimab, imdevimab, casirivimab, tixagevimab, cilgavimab, and sotrovimab) has undergone sequential phases of obsolescence (Cho et al., 2023). In the realm of *in vitro* assessments pertaining to antibody activity, sotrovimab’s capacity for neutralizing BA.5 is found to be attenuated, denoted by an EC50 of 858.2 ng/ml. In contrast, Cilgavimab and Evusheld demonstrate EC50 values of 23.5 and 56.6 ng/ml, respectively, effectively reinstating the neutralization efficacy against BA.5. Additionally, a 500 mg dosage of sotrovimab retains a measure of partial neutralization activity against BA.5. Nonetheless, the constrained *in vitro* and *in vivo* efficacy of the 300 mg Evusheld dosage in the context of BA.5 neutralization prompts the recent FDA recommendation to transition to a 600 mg dosage. The majority of antibodies, including bamlanivimab, estesevimab, casirivimab, txiagevimab, C135, C144, among others, exhibited negligible or no detectable neutralizing activity against the BA.5 subvariant (IC50 > 10 μg/mL) (Gruell et al., 2022). Comparatively, the neutralizing efficacy of imdevimab (IC50 3.392 μg/mL), sotrovimab (IC50 3.671 μg/mL), cilgavimab (IC50 0.085 μg/mL), DZIF-10c (IC50 8.638 μg/mL), and BRII-196 (IC50 0.820 μg/mL) was notably restrained. The BQ and XBB subvariants have now developed complete resistance to bamlanivimab. Results against Omicron XBB.1.5 for most monoclonal antibodies, namely sotrovimab, cilgavimab, bebtelovimab, and casirivimab, showed concentrations greater than 50,000 ng/mL, indicating that these antibodies may not be effective against XBB.1.5 in a clinical setting(Uraki et al., 2023b). Furthermore, the Evusheld approved for COVID-19 prevention exhibits total ineffectiveness against the emerging subvariants. This circumstance directly presents a significant quandary for the millions of individuals who demonstrate limited vaccine responsiveness and compromised immunity to COVID-19.

The presence of the N460K mutation on the BQ.1 and BQ.1.1 subvariants results in the annulment of neutralizing activity against NTD-SD2 and RBD class 1 monoclonal antibodies, whereas the reduced potency of RBD class 3 monoclonal antibodies can be ascribed to the R346T and K444T mutations. Attributed to the additional R346T mutation in BQ.1.1 in comparison with BQ.1, the former demonstrates a heightened capability to evade antibodies of RBD class 3 mAbs, surpassing the latter in this regard. It is also noteworthy that BQ.1.1, XBB, and XBB.1 collectively exhibit the R346T and N460K mutations, signifying their proclivity to evade antibodies targeting these specific spike region epitopes throughout their evolutionary trajectory. The emergence of the Q183E mutation on XBB or XBB.1 leads to the loss of potency for C1520. Moreover, the N460K and F486S mutations confer resistance to RBD class 1 and 2 mAbs, respectively. Conversely, the acquisition of R346T, V455P, G446S, and F490S mutations engenders resistance against RBD class 3 mAbs (Wang et al., 2022b) (**Table 1**).

## Decreased susceptibility to antiviral drugs

Severe COVID-19 disease is closely related to cytokine storm and oxidative stress, especially in patients with comorbidities (Alam and Czajkowsky, 2022). Studies have shown that the severity of COVID-19 infection is closely related to the increased production of CRP and pro-inflammatory cytokines (IL-6, IL-17) (Daneshkhah et al., 2020; Huang et al., 2020), leading to an increased risk of pneumonia, ARDS, and heart failure. Finally, there is multiple organ failure. CRP and inflammatory cytokines (IL-6, IFN-γ) are weakened in hemodialysis patients after vitamin D (calcitriol) treatment (Shah Alam et al., 2021). Perhaps vitamin D is expected to reduce COVID-19 mortality by simultaneously enhancing the innate immune system to inhibit the production of cytokines and CRP, thereby reducing viral load and reducing overactivation of the adaptive immune system. ROS are usually produced as natural by-products of oxygen metabolism and play an important role in cell signaling (Baqi et al., 2020). Oxidative stress is a non-specific pathological state that reflects an imbalance between ROS production rate and antioxidant processes (Laforge et al., 2020). However, in SARS-CoV-2 infection and metabolic diseases such as hypertension, diabetes, and obesity, ROS levels increase dramatically and damage cellular structures, which directly leads to increased disease severity and mortality. Therefore, therapeutic intervention targeting oxidative stress is needed, such as the use of antioxidants to mitigate oxidative stress-induced cellular damage. In addition, targeting viral replication of SARS-CoV-2 is also a focus of therapeutic intervention. A variety of antiviral drugs have been used in clinical treatment, and new antiviral drugs are constantly being developed and used in clinical practice.

The category of small molecule antiviral drugs has received FDA approval for addressing SARS-CoV-2 infections, includes remdesivir, nirmatrelvir, and molnupiravir. On November 22, 2021, the FDA sanctioned Pfizer’s novel drug, Paxlovid—a composite formulation of nirmatrelvir and ritonavir. This drug gained approval for treating mild to moderate SARS-CoV-2 infections in adults, children, and individuals characterized by an elevated susceptibility to severe disease (Bartoletti et al., 2022). Nirmatrelvir is an orally available protease inhibitor that exerts its effect by cleaving two viral peptides, while Ritonavir is a potent inhibitor of cytochrome P450 (CYP) 3A4 enzymes. The combination use of Nirmatrelvir and Letermovir can enhance the concentration of Nimartevir within the intended therapeutic range. The risk of COVID-19 rebound infection and symptoms within 2-8 days after Paxlovid treatment for BA.5 was found to be higher than that for BA.2.12.1 (Wang et al., 2022a). Another research study undertook an evaluation of the IC50 values about remdesivir, molnupiravir, and nirmatrelvir against the BA.5 strain, manifesting an incremental reduction in the strain’s responsiveness to remdesivir, molnupiravir, and nirmatrelvir (Takashita et al., 2022). The susceptibility of XBB.1.5 to these antiviral drugs is similar to that of Omicron BA.2. These results indicate that remdesivir, molnupiravir, nirmatrelvir, and ensitrelvir are effective against XBB.1.5 and XBB *in vitro* (Cho et al., 2023). Currently, an array of studies postulates the potential therapeutic efficacy of small molecule antiviral agents. However, definitive validation through clinical trials remains pending. Significantly, an open-label clinical randomized controlled trial was executed in Shanghai, China, in April 2022, with the specific objective of rigorously assessing the therapeutic effects of Shufeng Jiedu Capsules (SFJDC) against the SARS-CoV-2 Omicron variant(Zhang et al., 2022). The findings revealed that, in contrast to the control group, the administration of Shufeng Jiedu Capsules (SFJDC) significantly amplified the patients’ rate of recovery (83.8% vs. 70.1%) and led to a reduction in the duration required for symptom resolution (4.9 vs. 5.9 days) (**Table 1**).

## T-cell immunity pertaining to the immune protection against various other SARS-CoV-2 Omicron subvariants

Cellular immunity stands as a fundamental facet of adaptive immune mechanisms, assuming a pivotal role in attenuating disease severity and orchestrating the regulation of pathogenic infections. This intricate interplay involves coordinated responses of CD4 and CD8 T cells (He et al., 2022; Lu and Yamasaki, 2022). The initiation of a helper T cell(CD4)response stands as an indispensable prerequisite for the maturation of a robust antibody response and a potent cytotoxic CD8 T cell response (Lu and Yamasaki, 2022). The infection caused by SARS-CoV-2 demonstrates intricate associations with a broad range of disease outcomes, where changes in T-cell responses may contribute to the observed diversity. Breakthrough infections frequently manifest as relatively mild symptoms, thus predominantly preserving the protective effectiveness against severe illness. Comparative insights from other respiratory conditions, such as influenza, likewise propose a pivotal role of T-cell responses in attenuating disease severity.

Administering an additional dose of the inactivated CoronaVac vaccine as a booster has demonstrated a favorable impact on T-cell responses. Individuals who received the initial inactivated vaccine followed by an mRNA-based booster have also exhibited an enhancement in their T-cell responses (Lim et al., 2022). Pozzetto et al. also documented that in comparison to homologous BNT162b2 vaccination, the utilization of heterologous ChAdOx1-BNT162b2 vaccine presents an escalated efficacy in counteracting infection (Pozzetto et al., 2021). In contexts where booster doses encompassed mRNA-1273, Ad26.CoV2.S, or BNT162b2, individuals initially immunized with Ad26.CoV2.S and subsequently administered mRNA boosters exhibited the most potent antibody and CD8 T-cell responses. Across diverse immunization protocols, mRNA-1273 exhibited the most robust correlation with CD4 T-cell responses (Atmar et al., 2022). The implementation of ChAdOx1 nCoV-19 as the primary COVID-19 vaccination, succeeded by subsequent administration of either homologous ChAdOx1 booster or heterologous BNT162b2 or mRNA-1273 boosters, demonstrated effectiveness rates of 50%, 67%, and 79% respectively, in countering asymptomatic infections(Nordström et al., 2021). A comparable result was documented in a study conducted in Brazil (Cerqueira-Silva et al., 2022). A study conducted to evaluate the efficacy against asymptomatic infections revealed that the effectiveness of receiving one or two doses of ChAdOx1 is markedly reduced (Avila-Ponce De León et al., 2022). However, there is an observed improvement in efficacy when a ChAdOx1 dose is succeeded by an mRNA booster dose. The most optimal level of efficacy is evident with the administration of a three-dose regimen of mRNA vaccines (Accorsi et al., 2022). mRNA-1273 provided the largest boost in T cell responses, both in subjects <70 y old and in those >70 y old (Sette et al., 2023).

Tfh cells are important for neutralizing antibody responses, and higher frequencies of circulating Tfh (cTfh) cells were associated with milder COVID-19 (Stephenson et al., 2021). In a study of convalescent subjects (Nelson et al., 2022), mild disease was associated with SARS- A higher proportion of cTfh and Th1 cells were associated with CoV-2-specific cells, which in turn was associated with a sustained anti-spike antibody response after viral clearance. Furthermore, SARS-CoV-2 CD4 T cell responses predict the magnitude, breadth, and duration of subsequent neutralizing antibody responses (Balachandran et al., 2022). Conversely, the magnitude and quality of T cell responses correlate with neutralizing antibody titers following infection(Kroemer et al., 2022).

The predominant consensus derived from the existing dataset indicates that the protective efficacy of T-cell responses directed at SARS-CoV-2 mutant strains remains substantially unaltered, whereas the impact on NAb responses is more pronounced, particularly in the context of the Omicron variant. This aligns with earlier investigations concerning the Class I and Class II T-cell epitopes of SARS-CoV-2, suggesting that the virus faces a paradox in concurrently generating a repertoire of epitopes sufficient to evade recognition by T cells on a population level while upholding its virulence. Nevertheless, these observations do not negate the potential for mutation-associated modifications to facilitate escape from recognition by specific individual HLA/epitope configurations(Sette et al., 2023). Recent research is indicative of the emerging phenomenon wherein the L452Q mutation exhibits the potential to induce immune evasion against the antiviral effects activated by HLA-A24 restricted cytotoxic T lymphocytes (CTLs) (Kimura et al., 2022a).

Current studies have shown that immunization with the Spike-XBB.1.5 vaccine can effectively stimulate T cell responses against RBD-XBB.1.5 and RBD-BA.5 antigens, suggesting its potential in activating general cellular immunity against the Omicron variant. On the other hand, other vaccines targeting early Omicron strains (spike-BA.5, spike-BF.7, and spike-BQ.1.1) failed to elicit cytotoxic CD8 T cell responses against XBB.1.5. These findings suggest that the XBB.1.5 subvariant strain may evade the original humoral and cellular immunity, while the Spike-XBB.1.5 protein vaccine can effectively trigger this immune response. Therefore, the development of second-generation vaccines capable of inducing broad-spectrum cellular and humoral immunity is crucial in mitigating the potential impact of viral mutations in the future(He et al., 2023).

## Conclusions and prospects

In summary, the existing vaccine administration or booster doses do not exhibit prominent efficacy in preventing infection, but prior infections can effectively prevent breakthrough infections from Omicron BA.5 and similar subvariants. Additionally, the BQ and XBB subvariants have shown resistance to clinically approved therapeutic antibodies. Nevertheless, clinical cases of existing subvariants like Omicron XBB.1.5 are associated with milder symptoms, primarily including headache, fever, cough, and fatigue, with a lower mortality rate. Despite the limited efficacy of current vaccines in preventing infection and the relatively reduced protective effectiveness against subvariants like Omicron XBB.1.5, current research evidence indicates that they still provide good protection against severe illness, hospitalization risk, and post-infection mortality. Therefore, vaccination remains more effective than acquiring immunity through infection, helping to mitigate the severe impacts of the virus. The number of RBD mutations in Omicron subvariants has an inverse correlation with NAb titers. Previous studies also have demonstrated that Spike XBB.1.5 vaccination can induce robust and comprehensive immune responses against XBB.1.5 and other prevalent subvariants of Omicron. Hence, the development of a universal vaccine with additional RBD mutations could be a crucial approach to prevent the disease from spreading or resurging further. Additionally, wearing masks, maintaining social distance, and practicing frequent hand hygiene continue to be highly effective preventive measures in controlling subvariants like Omicron XBB.1.5. In the future, we should persist with normalized epidemic prevention and control measures, prepare medical resources in advance, expedite the entire vaccine process, accelerate the development of vaccines and specific treatments for new variants.

## Author contributions

ZL: Writing – original draft, Writing – review & editing. JL: Writing – original draft, Writing – review & editing. SP: Writing – original draft, Writing – review & editing. YL: Writing – original draft, Writing – review & editing. CL: Writing – review & editing. JZ: Writing – review & editing. DW: Writing – original draft. RC: Writing – original draft. JS: Supervision, Writing – review & editing. KC: Funding acquisition, Project administration, Resources, Supervision, Validation, Visualization, Writing – review & editing.
