# Supplementary material for: An updated review of epidemiological characteristics, immune escape, and therapeutic advances of SARS-CoV-2 Omicron XBB.1.5 and other mutants
Source: Front Cell Infect Microbiol. 2023 Dec 14;13:1297078. doi: 10.3389/fcimb.2023.1297078 (PMC10752979; doi:10.3389/fcimb.2023.1297078)
Supplement: Supplementary file 1 [file Table_1.docx]

**Table S1.** Analysis of the role of the main mutation sites of SARS-CoV-2.

| SARS-CoV-2 | Mutation site | The effect of mutation site on protein function | References |
| --- | --- | --- | --- |
| WT | G614G | It increased the viral load in the upper respiratory tract of COVID-19 patients and may increase transmission. | (Plante et al., 2021) |
| Delta | L452R | It may lead to enhanced ACE2 binding and could affect the neutralizing activity of certain monoclonal antibodies. | (Motozono et al., 2021) |
|  | T478K | It may lead to enhanced ACE2 binding, enhancing the adaptability of mutant strains. | (Di Giacomo et al., 2021) |
| Omicron BA.1 | E484A | Conferred resistance to monoclonal antibodies. | (Pastorio et al., 2022) |
|  | N501Y | Significantly enhanced affinity for binding to ACE2. | (Liu et al., 2022) |
| Omicron BA.2 | T376A | less efficient spike cleavage | (Hu et al., 2022) |
|  | D405N | Reduced the efficacy of certain monoclonal antibodies. | (Selvavinayagam et al., 2022) |
| Omicron BA.5 | F486V | Conferred sites for antibody escape and sensitivity to binding of RBD-directed monoclonal antibodies. | (Tuekprakhon et al., 2022) |
| Omicron BF.7 | R346T | It may lead to enhanced ACE2 binding, enhancing the adaptability of mutant strains. | (Chenchula et al., 2023) |
| Omicron BQ.1 | K444T | Induced immune escape. | (Qu et al., 2023a) |
|  | N460K | Enhanced the neutralization resistance of the strain. | (Qu et al., 2023a) |
| Omicron BQ.1.1 | R346T | Induced immune escape. | (Qu et al., 2023a) |
| Omicron XBB.1.5 | F486P | It may lead to enhanced ACE2 binding, enhancing the adaptability of mutant strains. | (Qu et al., 2023c) |
